# A Retrospective Analysis of Provider-to-Patient Secure Messages: How Much Are They Increasing, Who Is Doing the Work, and Is the Work Happening After Hours?

**DOI:** 10.2196/16521

**Published:** 2020-07-08

**Authors:** Frederick North, Kristine E Luhman, Eric A Mallmann, Toby J Mallmann, Sidna M Tulledge-Scheitel, Emily J North, Jennifer L Pecina

**Affiliations:** 1 Division of Community Internal Medicine Department of Medicine Mayo Clinic Rochester, MN United States; 2 Mayo Clinic Rochester, MN United States; 3 Undergraduate Research Education Program Mayo Clinic Rochester, MN United States; 4 Department of Medicine NYU Grossman School of Medicine New York, NY United States; 5 Department of Family Medicine Mayo Clinic Rochester, MN United States

**Keywords:** patient messages, secure messages, patient portal, provider messages, electronic health records, electronic mail, communication, patients, physicians, physician assistants, nurse practitioners, nurses

## Abstract

**Background:**

Patient portal registration and the use of secure messaging are increasing. However, little is known about how the work of responding to and initiating patient messages is distributed among care team members and how these messages may affect work after hours.

**Objective:**

This study aimed to examine the growth of secure messages and determine how the work of provider responses to patient-initiated secure messages and provider-initiated secure messages is distributed across care teams and across work and after-work hours.

**Methods:**

We collected secure messages sent from providers from January 1, 2013, to March 15, 2018, at Mayo Clinic, Rochester, Minnesota, both in response to patient secure messages and provider-initiated secure messages. We examined counts of messages over time, how the work of responding to messages and initiating messages was distributed among health care workers, messages sent per provider, messages per unique patient, and when the work was completed (proportion of messages sent after standard work hours).

**Results:**

Portal registration for patients having clinic visits increased from 33% to 62%, and increasingly more patients and providers were engaged in messaging. Provider message responses to individual patients increased significantly in both primary care and specialty practices. Message responses per specialty physician provider increased from 15 responses per provider per year to 53 responses per provider per year from 2013 to 2018, resulting in a 253% increase. Primary care physician message responses increased from 153 per provider per year to 322 from 2013 to 2018, resulting in a 110% increase. Physicians, nurse practitioners, physician assistants, and registered nurses, all contributed to the substantial increases in the number of messages sent.

**Conclusions:**

Provider-sent secure messages at a large health care institution have increased substantially since implementation of secure messaging between patients and providers. The effort of responding to and initiating messages to patients was distributed across multiple provider categories. The percentage of message responses occurring after hours showed little substantial change over time compared with the overall increase in message volume.

## Introduction

### Background

The volume of secure messages in health care institutions is increasing. A major national survey in 2013 found that 29.6% of the US population had used the internet or email to communicate with a physician or a physician’s office in the previous 12 months [1]. More recently, Lee et al [2] found that 37% of customers of a pharmacy chain reported contacting their physicians by email in the last 6 months.

Several health care systems in the United States have examined the increase in the number of messages from patients. Crotty et al [3] noted a tripling in messaging from 2001 to 2010. Cronin et al, Masterman et al, and Shenson et al [4-6] showed dramatic increases in message volumes across multiple specialties, including surgical and pediatric specialties.

Providers have mixed feelings about secure messages. In a survey of 43 clinicians across 5 clinics, 63% disagreed with the statement, “secure messaging reduces my workload,” and 33% agreed that “secure messaging has a negative effect on my workflow” [7]. However, 61% agreed that “secure messaging has a positive effect on patient-clinician communication” [7].

### Objectives

With providers responsible for an increasing number of secure messages, we looked at how secure messages to patients are distributed among staff at a large health care institution. In addition, with the increasing workload of secure messages, we examined whether there were potential *work after work* issues of using time after normal work hours to complete message responses [8].

## Methods

### Setting

The study took place at Mayo Clinic, Rochester, Minnesota, United States. Mayo Clinic is a multispecialty clinic with more than 1 million visits annually. There are more than 2200 physicians and scientists at the Rochester, Minnesota, campus.

Mayo Clinic started using Patient Online Services (POS; a secure patient portal) in 2010 for the primary care practice in Rochester, which serves a population of about 140,000. The Mayo Clinic specialty practice started using POS in 2013. The Mayo Clinic specialty practice serves the local community of Rochester and takes referrals from other practices, both nationally and internationally.

The patient portal (POS) at Mayo Clinic gives patients the ability to view their laboratory results, radiology reports, medical images, office and hospital notes, and specialty consultations. In addition, POS has messaging capability for patients and providers to communicate asynchronously by sending messages through a secure server, which also sends these messages to the electronic health record (EHR). Patients must log in securely to POS to send a message. When providers initiate a message or respond to a patient-initiated message through POS, patients are notified by email. To protect privacy, the email notifying the patient of a provider message states that new information is available on their POS (portal) account. In the notification email, patients are given a link to the Mayo POS, but they still need to securely log in to their personal account to view and send these messages. These asynchronous POS messages between patients and providers are what we call secure messages.

Physicians, nurse practitioners (NPs), physician assistants (PAs), and registered nurses (RNs) can receive and respond to secure messages at Mayo Clinic. In addition, licensed practical nurses (LPN) and secretarial staff can also respond to and send secure messages to patients. The categories used in this paper were physician, NP/PA (combined NPs and PAs), RNs, and other (LPN and secretarial).

### Provider Secure Message Data Collection

We collected all secure messages sent from the providers at Mayo Clinic, Rochester, from January 1, 2013, to March 15, 2018. The secure message dataset contained the clinic number of the patient, the date and time of day the provider sent the message, the message text, and the identification code of the provider or provider group who sent the message. In addition, the dataset was dichotomously categorized by whether the provider message was a response to a patient-initiated secure message or a provider-initiated secure message. The provider-response message was defined as a reply to a patient message. Provider-initiated messages were messages to patients created de novo by the provider (or created with help from software, as explained below in Abstraction of Provider Messages for Content). The entire secure message dataset contained only mutually exclusive provider-initiated and provider-response messages.

### Patient Demographics

To examine the differences between patients who had provider-response messages during the initial time frame and those several years later, we examined demographics of the patients who initiated messages during the first 6 months of the study (January 1, 2013, to June 30, 2013) and patients who initiated messages from the last 6 months (September 15, 2017, to March 15, 2018).

### Abstraction of Provider Messages for Content

From a sampling period of October 2017 to February 2018, we randomized and abstracted 1200 messages, 100 each from 12 categories: 3 types of providers (physician, NP/PA, and RN) split into 2 different practices (primary care and specialty), split further into 2 different message types (response and provider initiated). This gave us the 1200 randomized messages of 100 each in 12 categories (3 provider types multiplied by 2 practice types multiplied by 2 message types).

Across the 12 categories, we further dichotomously coded these as being automated or not. We categorized messages as *automated* if the content was completely software generated, such as reminders for screening mammograms and missed appointment notifications. There were other messages initiated that were not completely automated but did not have a personal message. For example, messages reaching out for specific laboratory or imaging tests that required provider input to order, but the message was not personalized to the point of explaining any details about the purpose of the tests. An example of this would be “Your provider has requested the following testing: fasting blood work in March. Please respond with your availability through Patient Online Services.” These message types were also categorized as *automated*. Some messages from patients contained only an update that merely required an acknowledgment such as “thanks for the update.” When abstracting the content, we also categorized the message responses as containing only an acknowledgment to account for these. Additional information collected was whether there was reference to having consulted another provider. This was to quantify the frequency at which messages could involve more than one provider. An example of this was a message from an NP/PA who wrote: “I spoke again to our C. diff specialist. He would like you to finish 10 days of vancomycin.” 

It should be noted that Mayo Clinic has a web-based knowledge system called Ask Mayo Expert, which has text-based content and care process algorithms to help with specific clinical questions. Ask Mayo Expert also gives a list of Mayo experts in specific areas. In this case, the NP/PA may have used Ask Mayo Expert to get the name of the Mayo expert in *Clostridium difficile* enteritis. It was outside the scope of this study to see how often providers were using resources such as Ask Mayo Expert (or other web-based resources) to answer patient questions.

### Portal Registration and Unique Face-to-Face Patient Visit Counts

Portal registration information was obtained from the Mayo Clinic connected care data. The number of unique patients seen during face-to-face visits was obtained from the Mayo Clinic administrative data.

### Work After Work Measure

From the date and time the secure message was completed, we determined whether the messages were sent during the usual business hours of 8 AM to 5 PM from Monday to Friday US central/daylight saving time.

### Statistical Analysis

We used JMP version 13.1 statistical analysis software (SAS) for the descriptive statistics as well as for analysis of variance for the differences between messages per patient by year. We used the Cochran-Armitage trend test to examine the trends in proportions, such as the proportion of messages answered outside of Monday to Friday from 8 AM to 5 PM. JMP version 13.1 was used to randomize the selection of messages for abstraction.

### Ethics

We excluded all messages from individuals who had not given research authorization. Mayo Clinic sites in Minnesota ask all patients for their research authorization, which is not specific to any individual study. This study was approved by the Mayo Clinic institutional review board (IRB 17-004807).

## Results

### Message Distribution by Practice Type

A total of 3,941,618 messages were sent by Mayo Clinic providers between January 1, 2013, and March 15, 2018, associated with 353,177 unique patients. We excluded 6.06% (238,870/3,941,618) of the messages from patients who had not given research authorization. After exclusion of the patients without research authorization there were 326,805 unique patients to whom Mayo Clinic providers sent 3,702,748 messages over the study duration. Provider responses to patient-initiated messages accounted for 48.87% (1,809,614/3,702,748) of the messages; provider-initiated messages accounted for 51.13% (1,893,134/3,702,748). The primary care practice accounted for 28.31% (1,048,216/3,702,748) of the messages, and the specialty practices had 71.69% (2,654,532/3,702,748) of the messages. Primary care providers initiated 18.28% (676,674/3,702,748) of the messages and responded to 10.03% (371,542/3,702,748). Specialists initiated 32.85% (1,216,460/3,702,748) and responded to 38.84% (1,438,072/3,702,748).

### Practice Volumes and Portal Registration Over Time

The increase in message counts could not be explained by a large growth in patient visits. In fact, the number of unique patients with face-to-face visits each year remained relatively stable, from 365,943 in 2013 to 388,707 in 2017 resulting in a 6% increase. Portal registration in patients with appointments increased from 33% in 2013 to 62% in 2018.

### Patient Demographics

Table 1 shows the demographics of the patients who initiated secure messages and had provider responses. The primary care population started portal messages in 2010, whereas specialty practice started in 2013. At least twice as many patient-initiated messages were sent by female patients in both the primary care and specialty practices in 2013, and this female predominance persisted into 2018. Older age groups, especially those ≥65 years, comprised an increasing proportion of the provider-response messages in 2018 compared with 2013.

**Table 1:** Demographic comparisons of the patients who initiated messages and had provider responses from 2013 to 2018 by provider type (primary care or specialty).

Demographic		Primary care response messages							Specialty response messages				
		First 6 months (2013; n=14,151), n (%)		Last 6 months (2017-2018; n=53,931), n (%)		*P* value^a^		First 6 months (2013; n=20,109), n (%)		Last 6 months (2017-2018; n=115,725), n (%)		*P* value^a^	
**Age group (years)**													
	0-17		443 (3.1)		4094 (7.6)		<.001		926 (4.6)		8095 (7.0)		<.001
	18-34		2951 (20.9)		9380 (17.4)		<.001		4555 (22.7)		16,730 (14.5)		<.001
	35-49		3769 (26.6)		13,796 (25.6)		.01		5473 (27.2)		23,542 (20.3)		<.001
	50-64		4903 (34.6)		15,861 (29.4)		<.001		6191 (30.8)		35,721 (30.9)		.82
	65-79		1801 (12.7)		8772 (16.3)		<.001		2592 (12.9)		27,220 (23.5)		<.001
	≥80		278 (2.0)		2023 (3.8)		<.001		360 (1.8)		4390 (3.8)		<.001
**Sex**													
	Female		9970 (70.5)		34,011 (63.1)		<.001		13,670 (68.0)		65,798 (56.9)		<.001
	Male		4175 (29.5)		19,915 (36.9)		<.001		6427 (32.0)		49,898 (43.1)		<.001
**Race**													
	White		13,182 (93.2)		49,424 (91.6)		<.001		18,574 (92.4)		105,646 (91.3)		<.001
	Asian		390 (2.8)		1655 (3.1)		.05		535 (2.7)		2614 (2.3)		<.001
	Black		151 (1.1)		709 (1.3)		.02		207 (1.0)		1449 (1.3)		.008
	Other		428 (3.0)		2143 (4.0)		<.001		793 (3.9)		6016 (5.2)		<.001
**Ethnicity**													
	Hispanic or Latino		199 (1.4)		975 (1.8)		.001		308 (1.5)		2255 (1.9)		<.001
	Not Hispanic or Latino		13,641 (96.4)		51,382 (95.3)		<.001		19,102 (95.0)		108,097 (93.4)		<.001
	Unknown/not disclosed		311 (2.2)		1574 (2.9)		<.001		699 (3.5)		5373 (4.6)		<.001
**Highest level of education**													
	Some postcollege graduate studies		2251 (15.9)		11,448 (21.2)		<.001		3526 (17.5)		28,092 (24.3)		<.001
	4-year college graduate		3752 (26.5)		9208 (17.1)		<.001		5183 (25.8)		21,866 (18.9)		<.001
	Some college or 2-year degree		2918 (20.6)		19,690 (36.5)		<.001		4155 (20.7)		37,619 (32.5)		<.001
	High school graduate		1454 (10.3)		6003 (11.1)		.004		2127 (10.6)		14,559 (12.6)		<.001
	Some high school (did not graduate)		234 (1.7)		1210 (2.2)		<.001		358 (1.8)		992 (0.9)		<.001
	Eighth grade or less		16 (0.1)		164 (0.3)		<.001		36 (0.2)		505 (0.4)		<.001
	Unknown		3526 (24.9)		6208 (11.5)		<.001		4724 (23.5)		12,092 (10.4)		<.001
**Patient’s home address**													
	Minnesota		13,059 (92.3)		52,663 (97.6)		<.001		14,196 (70.6)		68,538 (59.2)		<.001
	Contiguous state		357 (2.5)		651 (1.2)		<.001		2053 (10.2)		20,585 (17.8)		<.001
	Other (other US states and international)		735 (5.2)		617 (1.1)		<.001		3860 (19.2)		26,602 (23.0)		<.001

^a^Null hypothesis (H0): within primary care or specialty, first 6 months demographic proportion=last 6 months demographic proportion.

### Message Counts Over Time

**Figure 1:**
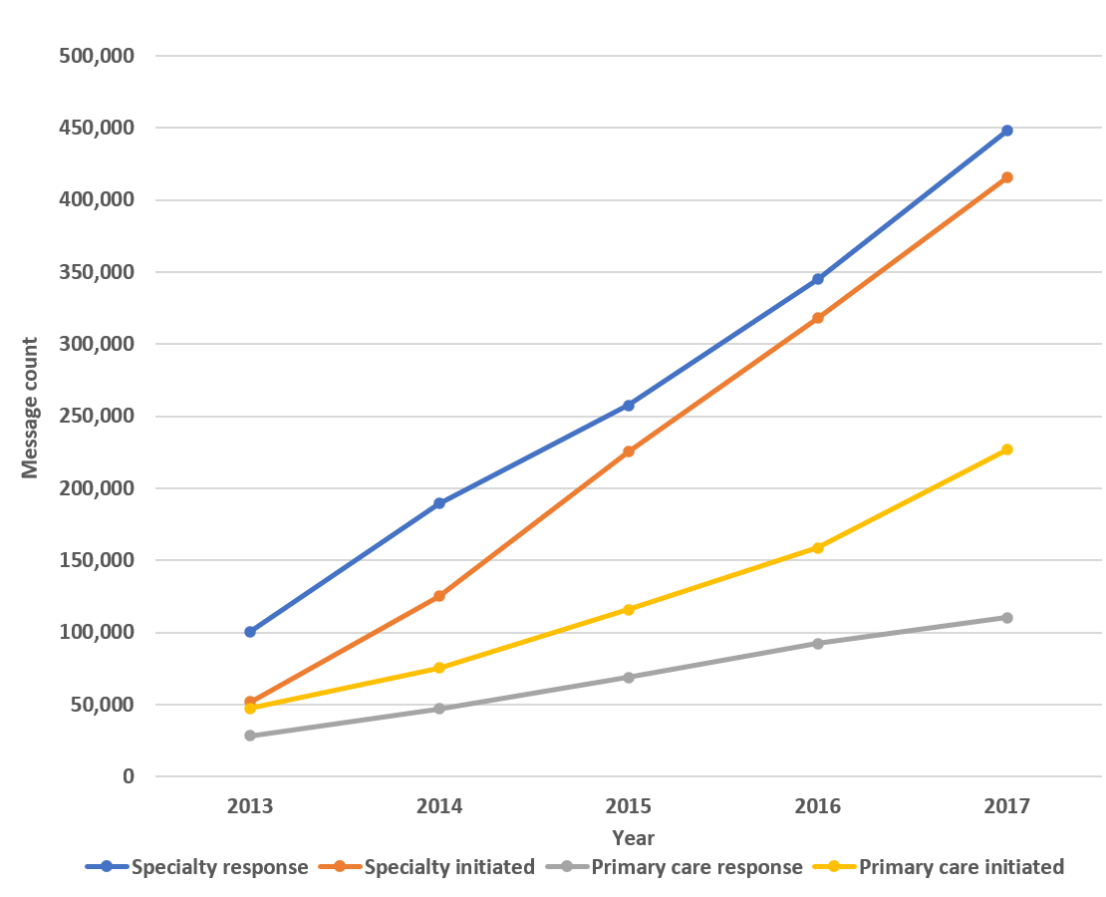
Message counts by year.

**Figure 2:**
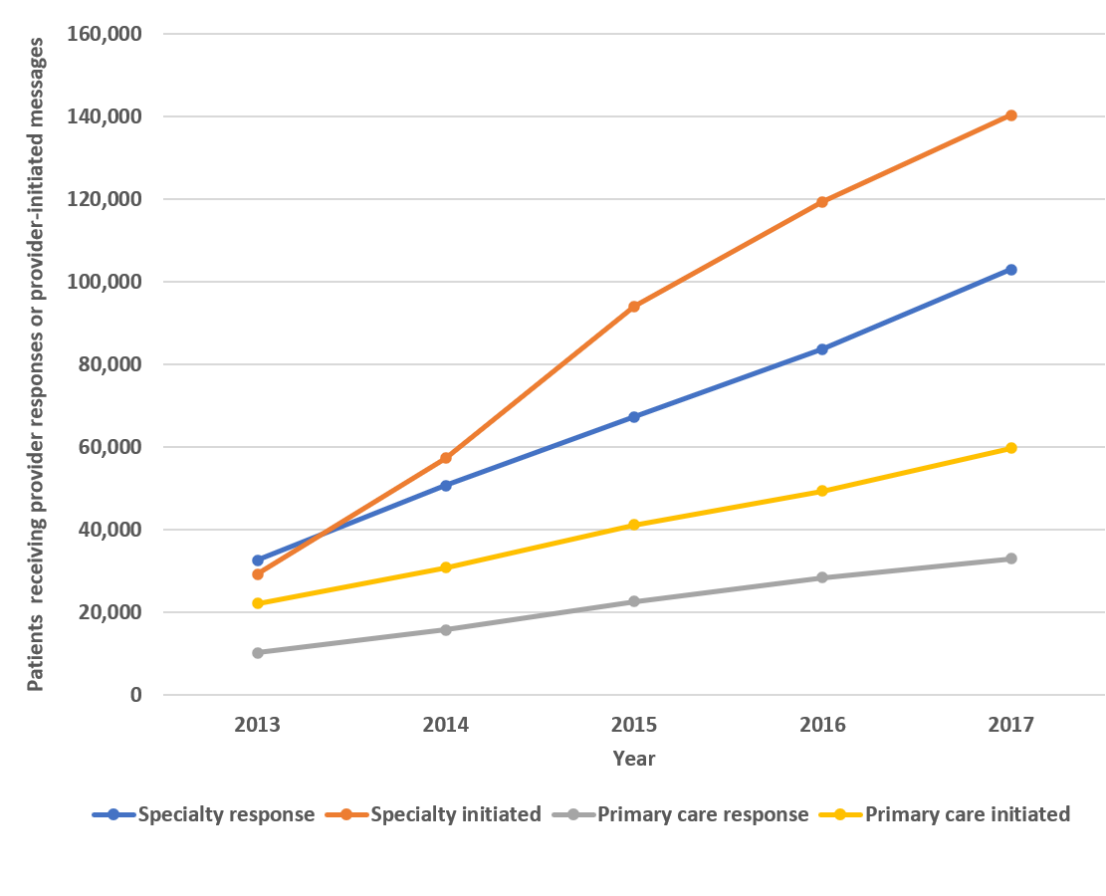
Distinct patients generating a provider response or provider-initiated message by year.

**Figure 3:**
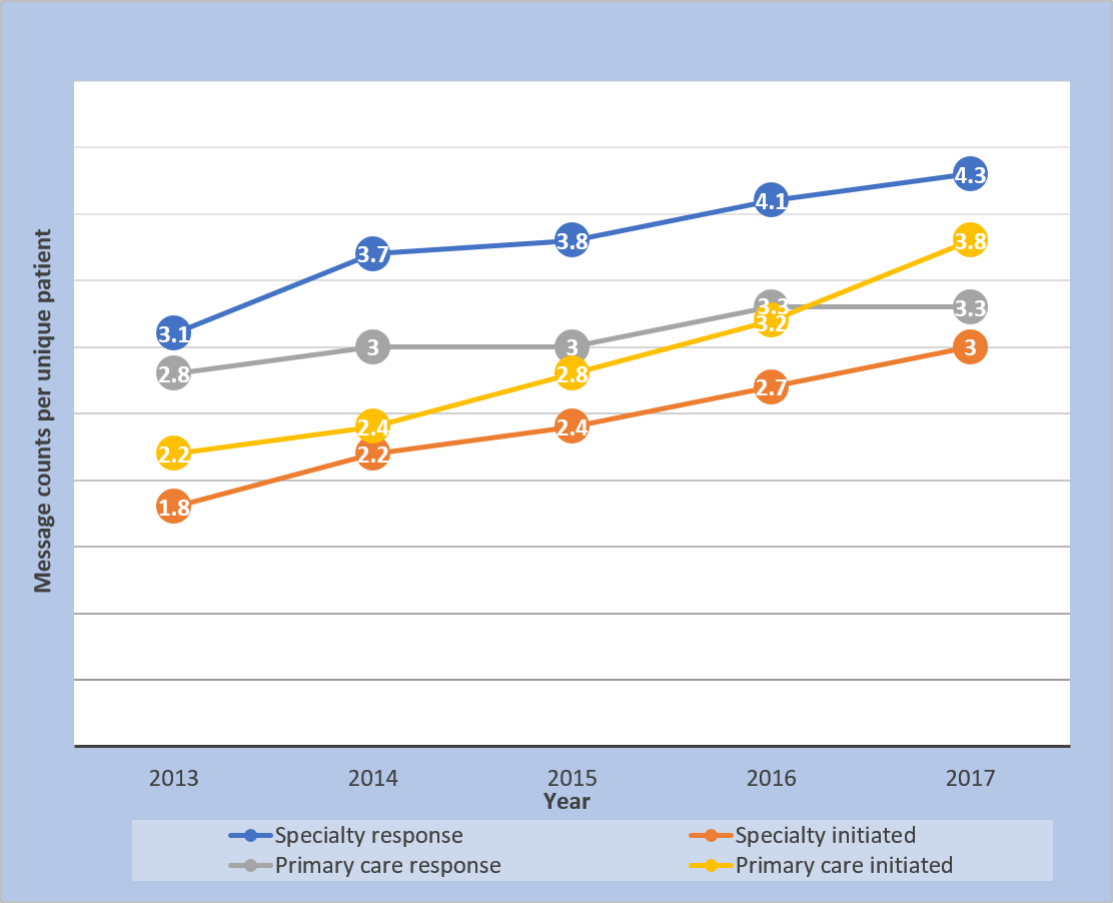
Message counts per unique patient by year.

**Figure 4:**
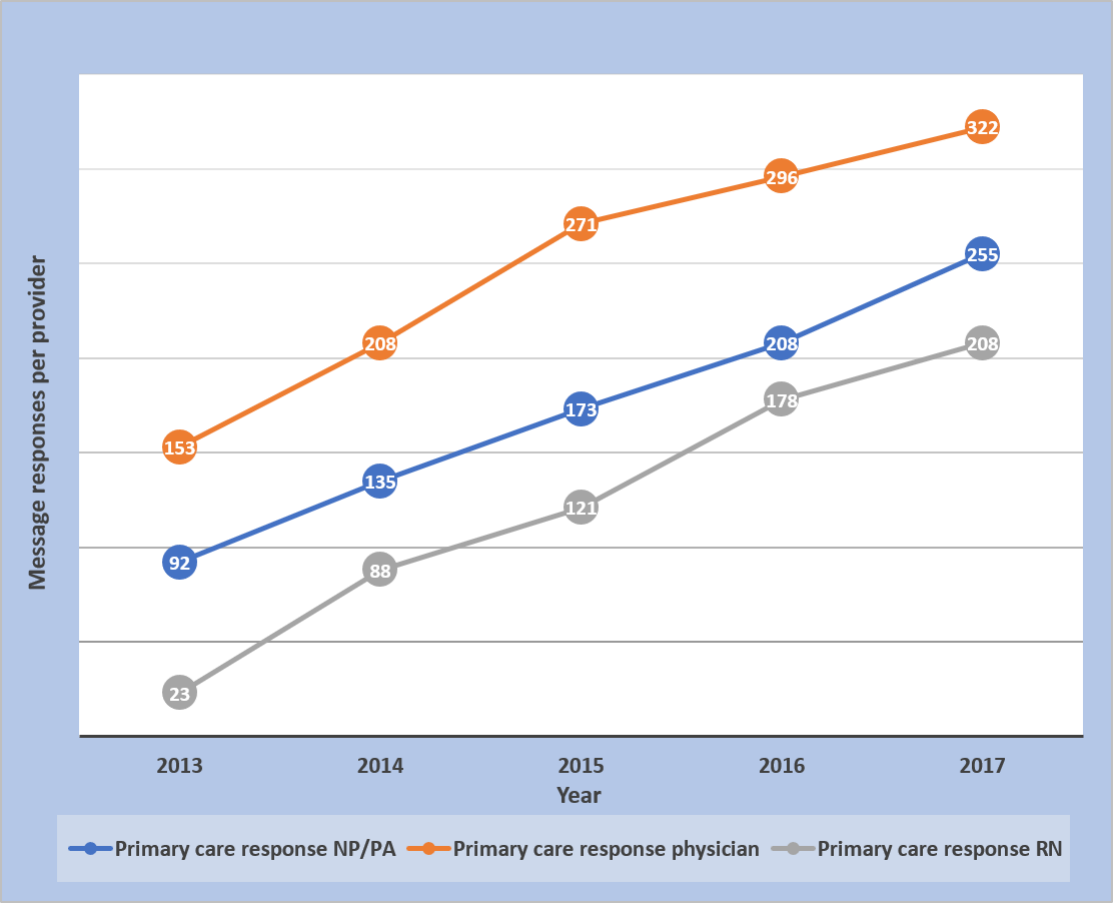
Message responses in primary care per provider by year.

**Figure 5:**
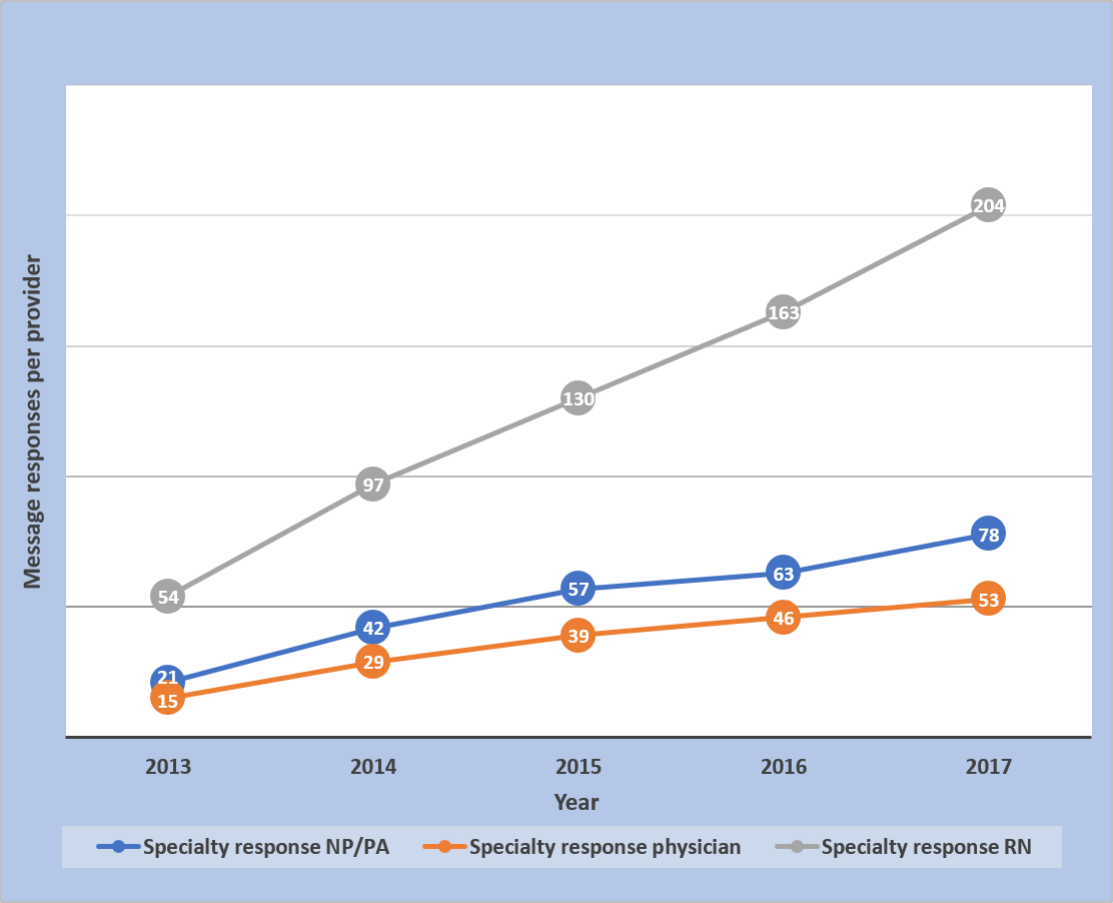
Message responses in specialty care per provider by year.

Figure 1 shows the increase in provider messages from 2013 through 2017. Figure 2 shows the rise in distinct patients who received provider messages over the course of the study. Messages per unique patient increased over the 5-year course of the study (Figure 3), and the increase in both provider response to messages and provider-initiated messages per unique patient was statistically significant (Table 2). Figures 4 and 5 show the number of message responses per provider by year for primary care and specialty care.

**Table 2:** Messages per unique patient by year.

Message type	Mean messages per unique patient by year (SD, 95% CI)						*P* value^a^
	2013	2014	2015	2016	2017		
Provider message responses from all specialty and primary care	3.6 (5.3, 3.5-3.6)	4.2 (6.6, 4.1-4.2)	4.3 (6.3, 4.3-4.3)	4.7 (6.7, 4.6-4.7)	4.9 (6.9, 4.9-4.9)	<.001	
Provider-initiated messages from all specialty and primary care	2.1 (2.0, 2.1-2.1)	2.5 (2.7, 2.5-2.5)	2.8 (3.1, 2.8-2.8)	3.2 (3.6, 3.2-3.3)	3.7 (4.1, 3.7-3.7)	<.001	

^a^H0: mean messages are equal across years.

Tables 3 and 4 show that the number of unique patients receiving provider message responses increased from 133% to 1215% across provider groups. Some of this increase could be attributed to more patients having access to secure messages. During the same 5-year interval, there was an 88% increase in portal registration (33% registered in 2013 to 62% in 2017). In contrast, the average number of provider-response messages per unique patient increased by at most 21% across provider groups. Nurses had the largest increases in messages per provider in both primary care (804%) and specialty care (278%). The percentage of messages completed after hours increased or decreased depending on the provider group, as shown in more detail (Tables 3 and 4). However, across all provider groups, there were more messages per provider completed after hours in 2017 than in 2013 (Tables 3 and 4).

**Figure 6:**
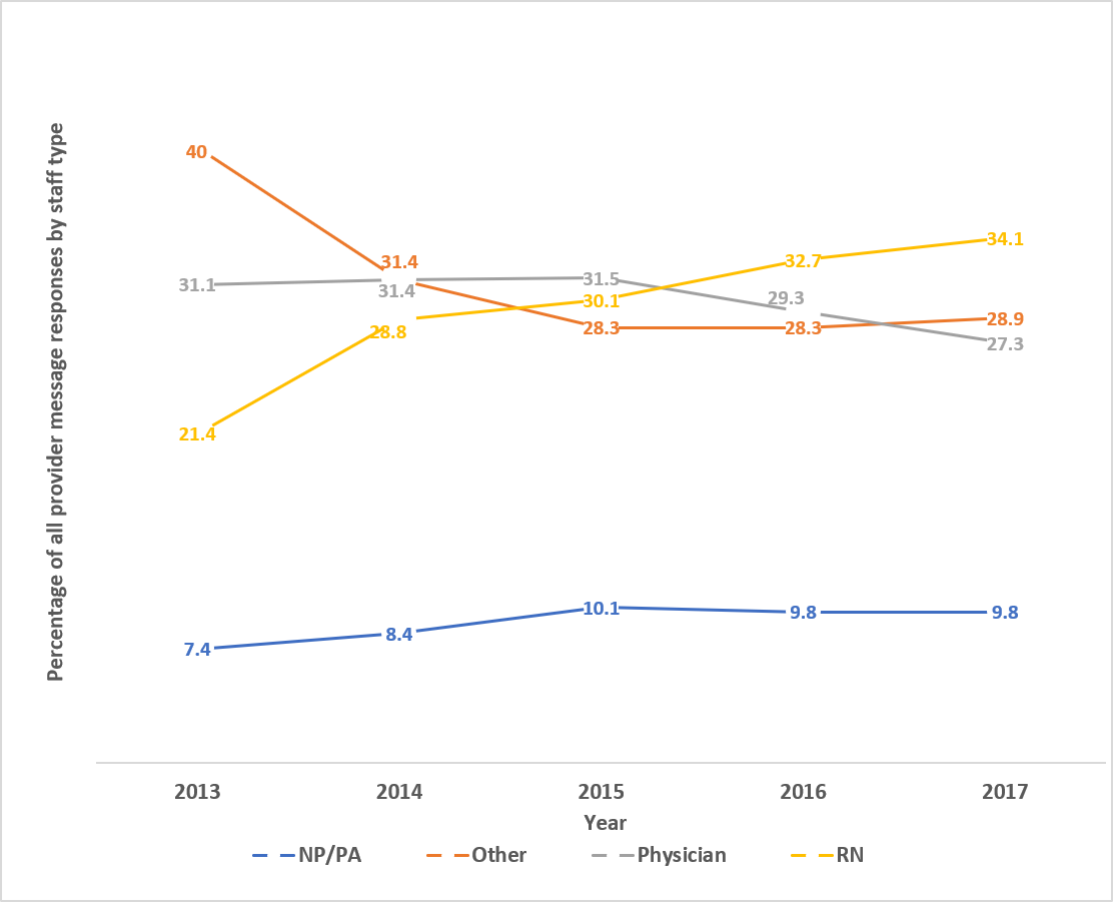
Percentage staff distribution responding to patient messages by year.

Figure 6 shows the change in work distribution for responding to messages over 5 years. Statistical analysis using the Cochran-Armitage test for trend for the data in Figure 6 showed that there were statistically significant downtrends in the percentage of message responses completed by physicians as well as *other* (*P*<.001). There were also statistically significant uptrends in the percentage of message responses completed by nurses and NP/PAs (*P*<.001).

**Figure 7:**
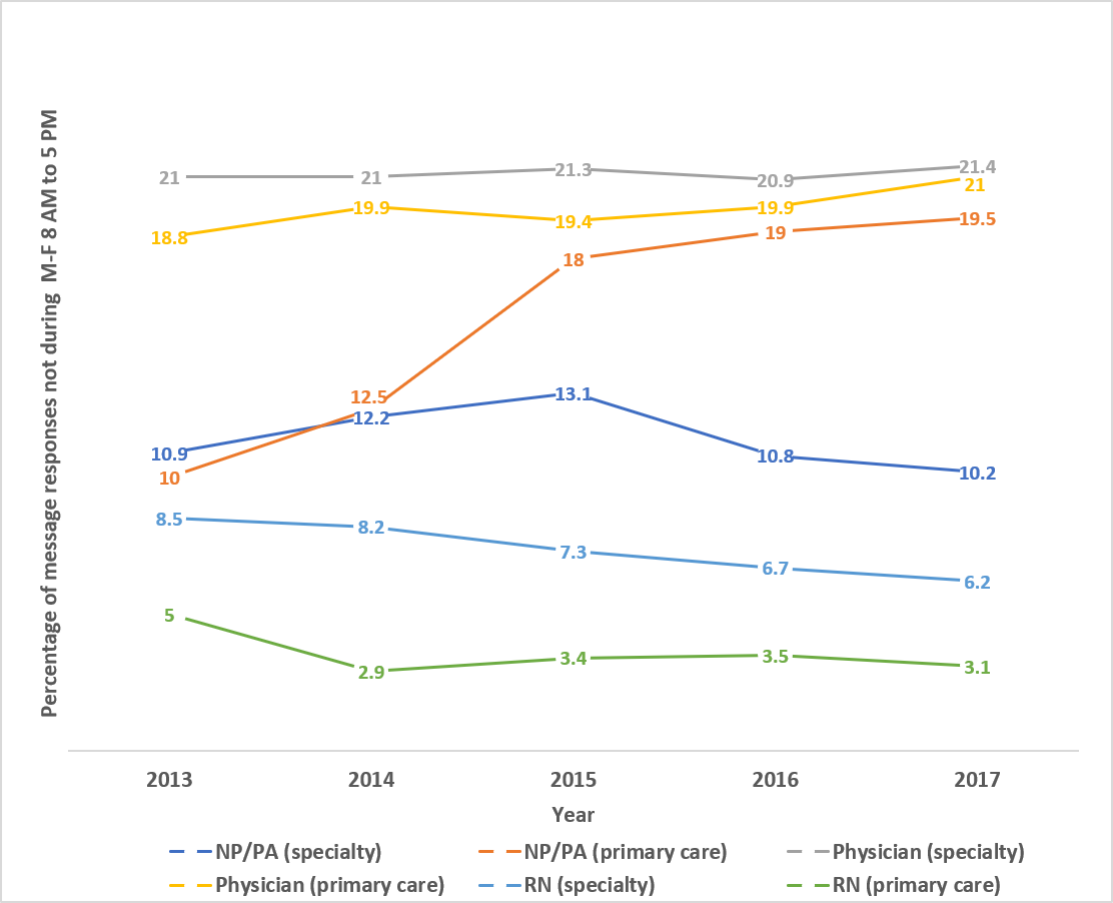
Percentage provider message responses outside of Monday-Friday 8 AM to 5 PM by year.

Figure 7 shows the percentage of messages completed after hours by staff type and year. Statistical analysis using the Cochran-Armitage test for trend of the data shown in Figure 7 showed that the percentage of after-hours message responses trended up for primary care physicians and primary care NP/PAs from 2013 to 2018 (each with *P*<.001). There was no significant trend in after-hours specialty physician responses and primary care RN responses (*P*=.10 and *P*=.11, respectively). There was a significant downtrend for after-hours RN specialty responses and NP/PA specialty responses (each with *P*<.001). It should be noted that Mayo Clinic has salaried physicians, NP, and PA staff and does not base salary or any other compensation on the numbers of secure messages answered or initiated, whether during or after hours. However, for hourly compensated staff in nursing and other nonphysician/NP/PA staff, overtime work would be compensated.

**Table 3:** Primary care messages.

Message category and year		Message count	Unique patients	Provider count	Messages per patient	Messages per provider	Percentage completed after hours, %	Messages per provider completed after hours
**Physician responses to messages**								
	2013	20,299	7441	133	2.73	153	18.8	29
	2017	47,700	17,323	148	2.75	322	21.0	68
	Change (%)	+135	+133	+11	+1	+110	+11.6	+134
**NP^a^/PA^b^responses to messages**								
	2013	4864	2105	53	2.31	92	10.0	9
	2017	25,772	11,572	101	2.23	255	19.5	50
	Change (%)	+430	+450	+91	−3	+177	+94	+456
**RN^c^ responses to messages**								
	2013	2053	1261	89	1.63	23	5.0	1
	2017	32,904	16,587	158	1.98	208	3.1	7
	Change (%)	+1503	+1215	+78	+21	+804	−38	+600
**Physician-initiated messages**								
	2013	36,093	17,691	133	2.04	271	N/A^d^	N/A
	2017	147,020	44,049	153	3.34	961	N/A	N/A
	Change (%)	+307	+149	+15	+64	+255	N/A	N/A
**NP/PA-initiated messages**								
	2013	9095	2105	47	4.32	194	N/A	N/A
	2017	54,872	9266	89	5.92	617	N/A	N/A
	Change (%)	+503	+340	+89	+37	+218	N/A	N/A
**RN^d^-initiated messages**								
	2013	2119	1063	57	1.99	37	N/A	N/A
	2017	18,278	11,039	160	1.66	114	N/A	N/A
	Change (%)	+763	+938	+181	−17	+208	N/A	N/A

^a^NP: nurse practitioner.

^b^PA: physician assistant.

^c^RN: registered nurse.

^d^N/A: not applicable. The percentage completed after work was not applicable in the provider-initiated messages because of the high percentage of automation.

**Table 4:** Specialty care messages.

Message category and year			Message count		Unique patients		Provider count		Messages per patient		Messages per provider		Percentage completed after hours, %		Messages per provider completed after hours
**Physician responses to messages**															
	2013	19,919		7677		1337		2.59		15		21.0		3	
	2017	104,624		36,825		1989		2.84		53		21.4		11	
	Change (%)	+425		+380		+49		+10		+253		+2		+267	
**NP^a^/PA^b^responses to messages**															
	2013	4715		2103		229		2.24		21		10.9		2	
	2017	28,668		12,542		369		2.29		78		10.2		8	
	Change (%)	+508		+496		+61		+2		+271		−6		+300	
**RN^c^responses to messages**															
	2013	25,635		7966		475		3.22		54		8.5		5	
	2017	157,478		42,892		772		3.67		204		6.2		13	
	Change (%)	+514		+438		+63		+14		+278		−27		+160	
**Physician-initiated messages**															
	2013	20,711		13,982		993		1.48		21		N/A		N/A	
	2017	189,997		92,503		2048		2.05		93		N/A		N/A	
	Change (%)	+817		+562		+106		+39		+343		N/A		N/A	
**NP/PA-initiated messages**															
	2013	5867		4382		143		1.34		41		N/A		N/A	
	2017	49,697		28,911		344		1.72		144		N/A		N/A	
	Change (%)	+747		+560		+141		+28		+251		N/A		N/A	
**RN-initiated messages**															
	2013	14,616		6458		341		2.26		43		N/A		N/A	
	2017	99,421		38,128		732		2.61		136		N/A		N/A	
	Change (%)	+580		+490		+115		+15		+216		N/A		N/A	

^a^NP: nurse practitioner.

^b^PA: physician assistant.

^c^RN: registered nurse.

^d^N/A: not applicable.

### Content Abstraction and Word Counts

In the primary care provider-initiated messages, automated messages accounted for 66%, 53%, and 18% of the physician, NP/PA, and nurse-initiated messages, respectively. For the specialty practice, automated messages accounted for 54%, 47%, and 13% of the physician, NP/PA, and nurse-initiated messages, respectively.

There were only a few provider-response messages that were limited to just an acknowledgment, such as “Thanks for the update.” These acknowledgment messages accounted for only 2%, 1%, and 1% of the primary care physician, NP/PA, and nurse response messages, respectively. Similarly, for specialty providers, brief acknowledgment messages accounted for 2%, 6%, and 3% of physician, NP/PA, and nurse response messages, respectively.

Our message content review revealed that provider-response messages sometimes included a reference to an additional provider who was involved in some way with the response. For the primary care providers, 2%, 1%, and 24% of the respective physician, NP/PA, and nurse responses had evidence of involvement of another provider in the message response. With the specialty providers, other providers were involved in 1%, 6%, and 43% of the responses from physicians, NP/PA, and nurse responses, respectively. It should be noted that in our samples of 100 provider-response messages, we found no automated responses in the physician, NP/PA, or nurse messages responding to patient-initiated messages.

The median word counts from provider responses did not vary much over the course of the study; they remained just over 70 words for both the primary care and the specialty practices. Some of the word count was a standard signature that contained some packaged terms thanking patients for using the portal.

## Discussion

### Principal Findings

Both specialty and primary care practices experienced a large increase in the number of provider message responses as well as the number of provider-initiated messages to patients. There was no single provider category that took the brunt of the message volume increase. In fact, the provider categories of physicians, NP/PAs, RNs, and *other* all shared in handling the responses to patient messages. All these provider categories also shared in the increased volume of provider-initiated messages.

The large increase in message volume was not because of increases in patient visits. Patient visits increased by <10%, whereas during the same time, provider responses to messages increased by 288% in the primary care practice and 345% in the specialty practice. The rise in responses to patient messages was also much greater than the 88% increase in portal registration during that time. These facts support the finding that there was increasing provider engagement in messaging during the course of the study. That is, an increasing proportion of providers were responding to and sending messages. However, the message volume was not just driven by more providers messaging their patients; there were increases in message volumes per provider across the board (Figures 4 and 5). Messages per patient also increased in both primary and specialty care practices (Figure 3 and Table 2). Hoonakker et al and Wolcott et al [7,9] reported that patient messaging was associated with providers initiating messages. Perhaps the increase in provider-initiated messages encouraged individual patients to engage more frequently in secure messages.

Implementation of secure messages was staggered, with primary care starting in 2010 and specialty practices in 2013. As a result, we had the opportunity to examine 62 months of secure message volumes for different time sequences. We observed specialty practice volumes for 62 months, starting from initiation in 2013 to 2018. As the primary care practice secure messages were implemented 3 years earlier (2010), the same 62 months encompassed years 4 to 9 of primary care message volumes. Over the same 62 months, but at different stages of experience with secure messages, secure message volumes showed continued growth both in primary care practice and in specialty practice. When separated into categories of provider message responses and provider-initiated messages, both categories showed a consistent rise over the 62 months.

### Work After Work

In an ideas and opinion paper in the *Annals of Internal Medicine*, DiAngi et al [8] described some novel metrics for examining EHR use. This included a *work after work* metric that “captures the hours the clinician spends logged into the EHR during evenings, weekends, and vacations.” The novel metrics also included what was termed *fair pay*, which are metrics that track “uncompensated EHR work, such as answering patient emails, providing medication refills...” [8]. Our study shows that approximately 20% of the time physicians completed message responses outside the usual business hours of 8 AM to 5 PM. There was a statistically significant uptrend over time for primary care physicians; their after-hours messaging increased from 19% to 21%, but not for specialty physicians whose after-hours messaging stayed stable between 21% and 22%. This finding is similar to that of Arndt et al [10], who found that approximately 24% of the EHR work done by physicians (1.4 hours out of 5.9) occurred after hours. NP/PAs in the specialty practice also used after-work hours for approximately 12% of their message responses across the study period. The largest work after work increase was in the NP/PA category in primary care, whose percentage of messages sent after work hours increased from 10% to 19%. The RN message responses, despite the increase in volume, had a statistically significant downtrend in percentage after-hours message completion.

As noted previously, physicians as well as the NP/PA staff are salaried, whereas a large number of nurses are on hourly wages. Although there was no statistically significant upward trend in percentage of message responses from specialty physicians completed after hours and only a couple of percentage points increase for primary care physicians, it remains that approximately 20% of these provider messages from physicians and primary care NP/PAs were sent after hours. There have been several papers associating EHR with burnout [11,12]. With the increase in secure messages, we thought that providers might be doing a larger percentage of the work after hours. Although there was a statistically significant increase in the percentage of after-hours messages completed by some provider groups, the rate of increase was low compared with the overall increase in the message volumes. However, because of the increase in message volume, all the provider groups completed more after-hours messages per provider in 2017 than in 2013 (Tables 3 and 4).

After data collection for this study was complete, Mayo Clinic switched to the Epic EHR. Epic has data collection methods that track message volumes and individual provider input (voice and keyboard) and can give management and providers feedback on the time spent in sending and receiving messages. This granular data about provider EHR activity throughout the day can be used to better identify the overall impact of messaging on provider workloads. The increase in provider secure messages shows the need for further investigations to examine the best practices in answering messages. In addition, the expanding role of secure messages needs to be considered in future studies of provider burnout.

In addition to continued examination of the work after work associated with secure messages, there is an opportunity to continue to assess how the increase in provider messaging may influence the tone of the messages. Hogan et al [13] evaluated some of the tone involved in these provider messages and noted that 25% of messages from health care team members appeared hurried. Newer informatics tools addressing sentiment analysis should help examine the content of portal messages and the sentiments associated with them [14].

### Practice Implications

We found a high rate of growth of secure messages not attributable to increased patient visits, and the secure message growth continued to rise several years after implementation in primary care and specialty practices. Previous studies both at Mayo Clinic and elsewhere have demonstrated some of the effects of secure messages on subsequent face-to-face visits and some safety aspects related to secure messages [15-21], but we do not know all the ways that secure messages can affect the health care system. If secure messages cause a decrease in an equivalent volume of more time consuming, nonreimbursable telephone communication and letter correspondence, the increase in secure message volume may be a marker of increased efficiency. However, at least one study has shown no impact of messages on telephone message volume [22]. There is a need for further studies examining all forms of patient-provider communication, including the cost of *telephone tag* and transcription for letter correspondence to obtain a more comprehensive picture of the economic impact of secure messages [23].

Our study showed that large numbers of providers and patients are engaging in secure messages. From the provider standpoint, our study shows that this includes not only physicians but also large numbers of other providers, including nurses, NP/PA staff, and other groups. Nurses had the highest increases in message responses per provider and responded to more messages per provider in the specialty practice than the specialty physician and NP/PA groups combined (Figure 5). Regarding the division of work among staff, it deserves reemphasis that our content review found 43% of the specialty nurse responses, and 24% of the primary care nurse responses had evidence of input from another provider. Laccetti et al [24], who studied cancer center secure messages, also found a sizable percentage of messages was handled by nurses (29%) and other nonphysician staff. As message volumes continue to rise, it will be important to efficiently divide the message responses among staff so that those that can be handled by nursing or other ancillary staff will not be sent to physicians. Cronin et al [25,26] at Vanderbilt have been working on automating the important job of classifying and triaging patient messages. With the participation of multiple levels of staff in messages, our study underscores the importance of further examination of how secure messages are being used and the potential importance of trainable *rules of engagement* for different staff categories responding to messages [27].

These messages represent a new avenue to access medical care. Secure messages can be more convenient than telephonic services, which often have circumscribed hours of operation and can interpose several call transfers and waits between providers, care teams, receptionists, and patients. In addition, for many institutions, including Mayo Clinic, patient secure messages are answered free of charge. Thus, for those with web-based access, secure messages can be an attractive alternative to access a provider. Hospitalized patients are also now accessing secure messages through inpatient portals. Initial studies have shown that secure messages to providers in the hospital environment have not been as highly used by patients as other inpatient portal features [28]. However, nurses and other hospital staff have seen benefits from the inpatient portal, and there is new insight on how to best introduce the inpatient portal to hospitalized patients [29,30]. Hospitalized patients who are introduced to the inpatient portal may be more likely to engage in secure messages after hospital discharge when nurses are more than a few steps away [28].

Automated messages were a large part of the provider-initiated messages; at this point, there is limited data concerning the impact of these messages and patient acceptance of them. In addition, there were provider-response messages that indicated that more than one provider was involved in the message response. It will be important to understand the work that goes into these message responses as newer forms of payment for services are being considered.

Our study shows the need to carefully examine the economics and outcomes of secure message responses in both accountable care and fee-for-service models. The increasing message volume comes in the context of a fixed number of work hours. As more time is spent with messages, there will be increasing pressure on face-to-face time, regardless of the payment system. The increase in messages is likely to cause significant outcomes and economic impacts with either payment model.

### Limitations

This study had several limitations. First, this was limited to one large multispecialty group located in North Central United States. The patients were mostly white and well educated, and the percentage of patients seen in the clinic who are registered on the portal increased to 62% over the course of the study. Our study also had a much higher rate of provider-initiated messages compared with a veteran’s health administration study by Shimada et al [31], which showed that only 5.5% of messages were initiated by providers proactively reaching out to patients.

As seen in Table 1, there were changes in demographics of patients who were sending messages from the initiation of the study (early users of secure messages) to 5 years later. We did not perform an in-depth analysis of the demographics of face-to-face Mayo Clinic patients simultaneously at the same time points. It is possible that some of the longitudinal shift in demographics that we saw in patients using messages could be confounded by a 5-year demographic shift of all Mayo Clinic patients.

Our experience at Mayo Clinic in Rochester, Minnesota, may not be generalizable to other multispecialty groups. Mayo Clinic has a specialty care focus, and patients both nationally and internationally come to Mayo Clinic to receive highly specialized care. As seen in Table 1, approximately 40% of specialty response messages went to patients who did not live in Minnesota. The geographical distance of many of the Mayo specialty patients may encourage a higher use of secure messages compared with other multispecialty groups whose patients have fewer geographic barriers to face-to-face visits.

LPNs were also involved in some message responses and initiated messages but accounted for only 0.7% (12,680) of message responses and 1.6% (29,774) of initiated messages, so they were put in the *other* category. The staff responding to and initiating messages in the *other* category were generally secretaries and clinical assistants involved in scheduling; some of these messages did not have an associated identifier that we could assign to either primary care or specialty care. In addition, there were small percentages of nurses and NP/PAs who transferred from specialty to primary care or vice versa; our administrative data could not correct for these individual changes. However, these changes were likely limited to the RN and NP/PA groups, as physicians were constrained by physician specialty board certification.

### Conclusions

In the first 8 years of secure message use in a large multispecialty group, secure message volumes showed large increases both in response to patient messages and provider-initiated secure messages. Both primary care practices and specialty practices saw large growth rates in total messages and messages per provider. Physicians, NP/PAs, and RNs all shared in responding to the increased volume of messages. Messages per unique patient also showed a significant increase over 5 years. The percentage of message responses after hours stayed close to 20% each year over 5 years for physicians in primary care and specialty practices.

## Abbreviations

EHR: electronic health record
LPN: licensed practical nurse
NP: nurse practitioner
PA: physician assistant
POS: Patient Online Services
RN: registered nurse
